# The Effect of Electroacupuncture on Dynamic Balance during Stair Climbing for Elderly Patients with Knee Osteoarthritis

**DOI:** 10.1155/2020/3563584

**Published:** 2020-08-13

**Authors:** Meijin Hou, Xiangbin Wang, Jiao Yu, Shengxing Fu, Fengjiao Yang, Zhenhui Li, Yanxin Zhang, Jing Tao

**Affiliations:** ^1^National Joint Engineering Research Centre of Rehabilitation Medicine Technology, Fujian University of Traditional Chinese Medicine, Fuzhou, Fujian 350122, China; ^2^Key Laboratory of Orthopaedics & Traumatology of Traditional Chinese Medicine and Rehabilitation (Fujian University of TCM), Ministry of Education, Fuzhou, Fujian 350122, China; ^3^College of Rehabilitation Medicine, Fujian University of Traditional Chinese Medicine, Fuzhou, Fujian 350122, China; ^4^Department of Rehabilitation Medicine, The First Affiliated Hospital of Fujian Medical University, Longyan, China; ^5^Department of Exercise Sciences, Faculty of Science, The University of Auckland, Auckland, New Zealand

## Abstract

**Background:**

Poor balance is one of the risk factors for falls in patients with knee osteoarthritis (KOA), which is related to the symptoms. Electroacupuncture (EA) is one of the traditional Chinese conservative methods commonly used to improve the symptoms in patients with KOA.

**Objective:**

To assess whether EA increases the dynamic balance during stair negotiation among patients with KOA.

**Methods:**

A total of 40 KOA patients were assigned to two groups randomly (true electroacupuncture vs. mock electroacupuncture). Acupoints around the knee were selected in the true electroacupuncture (TEA) group with electrical stimulation (2 Hz). In the mock electroacupuncture (MEA) group, about 2 cm next to the above acupoints, the needles were inserted superficially without electrical stimulation. All the participants received 11 sessions of stimulation treatment in three weeks. The primary outcome was margin of stability (MOS). Secondary outcomes included hip kinematics and kinetics as well as pain.

**Results:**

There was no significant difference between the two groups for self-reported pain (*p*=0.585). During ascent, there was no difference between groups in MOS value in both directions, which was the anterior-posterior (A/P) direction and medial-lateral (M/L) direction at initial contact and toe-off as well as the midstance in the gait cycle, and no difference for the hip kinematics and kinetics between the groups was detected (*p* > 0.05). For descent, at the toe-off event, the TEA group was more unstable as compared to the MEA group in the A/P direction (*p*=0.029) but not in the M/L direction, and the hip showed a larger internal rotator moment (*p*=0.049); at the midstance, the TEA group showed a lower abductor moment than the MEA group (*p*=0.003).

**Conclusions:**

Based on the assessment results from the chosen patients with KOA, the TEA did not demonstrate a significant effect in improving the dynamic balance during stair negotiation in comparison with the MEA. This finding does not support EA as a conservative treatment to improve the dynamic balance in such patients.

## 1. Introduction

Among people age 45 years or older, the overall prevalence of symptomatic knee osteoarthritis (KOA) was 8.1% [1]. Elderly people suffered a high risk of falls [2], and the fall-related injuries contributed to a huge economic burden [3]. Elderly patients with KOA were 54% more likely to experience a fall than those without KOA in the past year [4]. So, the economic burden of elderly KOA patients may be higher than those of their peers.

A recent systematic review shows poor balance is one of the risk factors for falls in KOA patients [5], which is related to symptoms such as pain, muscle weakness, and limitation to range of joint motion [6]. Balance refers to the stability to maintain the centre of mass (COM) over the base of support (BOS) [6], and it can be divided into static and dynamic balance. The clinical standing balance tests, such as the Step Test, Single Leg Stance Test, or laboratory-based measurements of standing balance, such as the centre of pressure (COP) velocity and Biodex score, show that KOA patients performed worse with regard to standing balance than healthy controls [7, 8]. For dynamic balance, it refers to the ability to maintain the COM on the BOS by considering the body position and velocity while doing some dynamic tasks [9], and KOA patients also performed impairments, such as lower Community Balance and Mobility Scale scores as compared to the healthy controls [10]. Conservative treatment, such as transcutaneous electrical nerve stimulation (TENS) simultaneously combined with local heat [11] and electrical stimulation combined with continuous passive motion [12], can relieve pain and potentially improve the dynamic balance in KOA patients during gait. Acupuncture can improve the symptoms and functional activities of KOA patients [13] and alter the gait control strategy toward a normal pattern to meet the demand of supporting the body [14].

But most studies focused on the gait and paid less attention to the effect of the conservative treatment on the dynamic balance during stair negotiation. Stair climbing is a common activity of daily life. The fall risk of stair walking is higher than that of flat walking, especially in stair descent [15]. A previous study used the extrapolated centre of mass (XCOM) method to evaluate the dynamic balance when KOA patients descended stairs and disclosed that older individuals showed reduced dynamic balance control as compared with young individuals [16]. Therefore, the aim of this study was to determine the efficacy of acupuncture combined with electrical stimulation (true electroacupuncture, TEA) and mock electroacupuncture (MEA) for dynamic balance in patients with KOA. We hypothesized that TEA would be better than MEA to improve the dynamic balance in KOA patients during stair climbing, especially in descent.

## 2. Methods

The study is a randomized controlled clinical trial (ChiCTR-IIR-16010284) whereby the ethical approval was obtained from the Affiliated Rehabilitation Hospital of Fujian University of Traditional Chinese Medicine Institutional Review Board (No. 2016KY-007-01), and each participant signed an informed consent form voluntarily.

### 2.1. Participants

Forty patients (7 males) aged from 48 to 79 with a diagnosis of bilateral symptomatic KOA, in accordance with the random number table, were assigned into an experimental group (TEA group) and a control group (MEA group).

All participants met the following inclusion criteria: (1) had been diagnosed by a physician with bilateral symptomatic KOA according to the clinical and radiographic criteria proposed by the American College of Rheumatology [17], (2) had both knees with grade 2 or 3 according to the standards of the Kellgren/Lawrence grade [18] by radiographic examination and(3) maintained the ability to walk and climb stairs without assistance.

Participants were excluded if they met any of the following exclusion criteria: (1) had asymptomatic KOA patients or other neuromusculoskeletal diseases that may affect gait and/or cognitive function, such as lower extremity injury, rheumatoid arthritis, neuropathic arthropathy, stroke, or fracture; (2) accepted intra-articular injection, such as a corticosteroid, in the previous two months; (3) accepted a total knee replacement or planned to have the surgery in the following year; and (4) underwent any biomedical treatment, such as medication, exercise programme or physical therapy, traditional Chinese Medicine therapy, and other rehabilitative therapy in the last week.

### 2.2. Treatment

**Figure 1:**
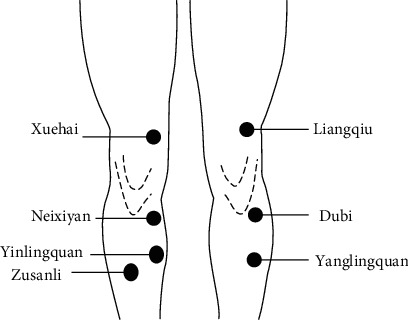
The location of acupoints.

Every participant of the experimental group accepted a 30-minute TEA treatment, while the control group received MEA treatment. According to a previous study [19] and the clinical experience of the members of the research group, seven acupoints around the knees, namely Neixiyan (EX-LE 4), Dubi (ST 35), Yanglingquan (GB 34), Yinlingquan (SP 9), Xuehai (SP 10), Liangqiu (ST 34), and Zusanli (ST 36) (Figure 1) were selected and acupunctured with a 1.5-inch single needle (0.30 × 40 mm, Wuxi Jiajian Medical Instruments Co., LTD, China) through the skin to a depth of 1.5 inch by an experienced acupuncturist. Then, the needles were connected to electric needling equipment (G6805, Shanghai Medical Instruments Co., LTD, China), of which the 2 Hz continuous square pulse at a maximal current intensity of patient tolerance was chosen. Each channel of G6805 has two electrodes, and we used two channels. One of the electrodes of the first channel was connected to the needle that was inserted into the Neixiyan, while the other one was connected to Yinlingquan. For the second channel, the electrodes were connected with Yanglingquan and Liangqiu, respectively. During the 30-minute TEA stimulation, all the patients reported “de qi” sensations, which is a feeling of heaviness, numbness, or distention at the area of acupoints [20]. In the MEA stimulation, the same needle as the experimental group was pierced into the skin at nearly 0.5 cm in depth at points that were 2 cm next to the acupoints and away from the meridian by another experienced acupuncturist. And the electric needling equipment was also connected to the needles with no power. During the 30-minute MEA stimulation, none of the patients had “de qi” sensations.

Both groups were treated for 3 weeks, which consisted of the first week of treatment once a day, continuous treatment of 5 days, and then followed by 2-day rest. The treatment of the second week and the third week were once every other day, three times a week. Finally, every patient underwent a total of 11 sessions of treatment.

### 2.3. Assessment

Before and after treatment, each participant was assessed for their knee pain using a 5-point Likert-type Western Ontario and McMaster Universities Osteoarthritis Index (WOMAC), where 0 means no pain, 4 means worst imaginable pain, and total score is 20. After the self-reported pain evaluation, all participants were asked to do the stair negotiation test using a three-dimensional gait analysis system (Motion Analysis, Santa Rosa, CA, USA) with a sampling rate of 100 Hz. Passive reflective markers with a diameter of 10 mm were placed on the skin according to the calibrated anatomical systems technique (CAST) protocol [21]: anterior superior iliac spine, posterior superior iliac spine, great trochanter, medial and lateral femoral epicondyle, medial and lateral malleolus, aspect of the Achilles tendon insertion on the calcaneus, dorsal margin of the first metatarsal head, dorsal aspect of the second metatarsal head, and dorsal margin of the fifth metatarsal head. Two force plates (AMTI BP400600, Advanced Mechanical Technology Inc., Watertown, MA, USA) were used to collect ground reaction force data with a sampling rate of 1000 Hz. The AMTI custom stairs [22] were fixed to the two force plate measure forces on each stair using a standard approach [23]. After familiarization with the test process, each participant performed a stand static trial and then five valid trials of stairs ambulation at a self-selected pace in the step-by-step manner without using the handrails. When participants felt tired, they had a brief rest between trials.

### 2.4. Data Analysis

Local segment coordinate systems were defined for the pelvis, thigh, shank, and foot segments based on markers' position using Visual 3D Version 6 (C-Motion Inc., Germantown, MA, USA). Joint kinematics were calculated using an *x*-*y*-*z* Cardan angle rotation sequence. Joint kinetics were calculated using inverse dynamics, and the joint moments were normalized to body mass (Nm/kg). Centre of mass (COM) was calculated using a standard procedure as described by David Winter [24], which was located in the pelvis.

Based on the data of COM displacement, the XCOM values were calculated as follows [25]:

(1) $$\begin{array}{c} \text{XCOM}={\text{COM}}_{\text{position}}+\frac{V_{\text{COM}}}{\sqrt{g/l}}, \end{array}$$

where COM_position_ refers the position of the COM in the anterior-posterior (A/P) and medial-lateral (M/L) direction, which was relative to the global coordinate system (GCS) of the LAB, *V*_COM_ was estimated by the A/P and M/L velocity of the COM, *g* is the gravity (9.8 m·s^−2^), and *l* was the distance between the COP and the COM.

The margin of stability (MOS) value was calculated as follows:

(2) $$\begin{array}{c} \text{MOS}=\text{BOS}-\text{XCOM}. \end{array}$$

As the position of the COM within the BOS while postural stability is sustained, a decrease in the MOS indicates that the XCOM exceeds the BOS and stability is therefore disturbed. In other words, larger values indicate greater stability, and negative values represent instability. In our study, we calculated the MOS in the sagittal plane (MOSAP) and the frontal plane (MOSML) using MATLAB 2016a (MathWorks Inc., Natick, MA, USA).

### 2.5. Statistical Analysis

For all the outcome parameters, namely pain scores, gait velocity and step width and hip joint angles and moments, as well as the MOS in two directions were extracted to do the statistical analysis using the SPSS statistical package Version 20 (SPSS Inc., Chicago, IL, USA), and all significant levels were set at *p* < 0.05. The normality of all outcome parameters was initially tested. The analysis of covariance totally has greater statistical power to detect a treatment effect than the other methods [26]. So, we choose the analysis of covariance (ANCOVA) to assess the differences between these two different treatment effects on KOA patients. Each outcome parameters used its own pretreatment data as the covariance.

## 3. Results

**Figure 2:**
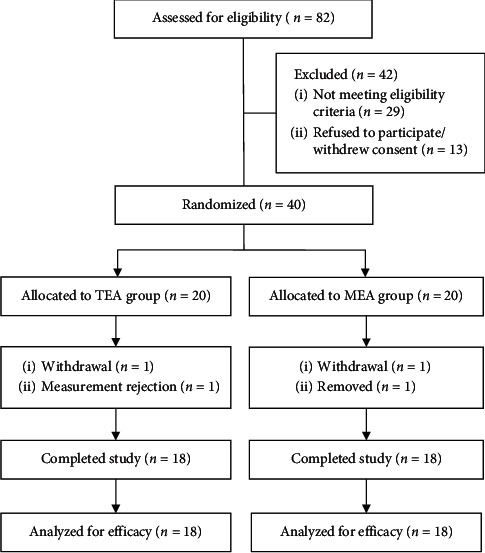
Flow chart of study procedure.

36 (6 males) patients finished the research. Four subjects did not finish the study due to a car accident (*n* = 1), scheduling (*n* = 1), measurement rejection (*n* = 1), and removal because of wrong treatment (*n* = 1). After the drop out of 4 subjects from the study, each group had 18 individuals (Figure 2).

There was no significant difference between the groups for age (61.11 ± 8.62 years for the TEA group vs. 65.94 ± 6.00 years for the MEA group, *p*=0.06), BMI (22.89 ± 2.47 kg/m^2^ vs. 24.61 ± 2.63 kg/m^2^, *p*=0.051), duration of KOA (103.42 ± 113.25 months for the TEA group vs. 96.67 ± 148.79 months for the MEA group, *p*=0.75), and the male-female ratio (4/14 vs. 2/16, *p*=0.37). For both study and control groups, the pain recorded from posttreatment was significantly improved in comparison with pretreatment. However, no statistically significant differences have been identified (Tables 1 and 2).

### 3.1. Ascent

The mean MOS value in both A/P and M/L directions during ascent for each group is shown in Table 1, and there was no between-group difference at initial contact (IC) and toe-off as well as the midstance (MS) in the gait cycle during ascent. After treatment, there was no significant difference between the groups for the velocity (0.52 ± 0.06 m/s vs. 0.51 ± 0.07 m/s, *p*=0.269) and step width (0.08 ± 0.03 m vs. 0.08 ± 0.03 m, *p*=0.434). Furthermore, no difference for the hip kinematics and kinetics between the groups was detected (*p* > 0.05) (Table 1).

### 3.2. Descent

After treatment, the velocity of stair descent showed no significant difference between the two groups (0.53 ± 0.05 m/s vs. 0.53 ± 0.08 m/s, *p*=0.273), with the TEA group walking faster than before. The same situation was shown in the step width (0.11 ± 0.03 m vs. 0.12 ± 0.04 m, *p*=0.549).  Table 2 also shows the details of the MOS value in both directions and the hip variables. At the initial contact, the MOS, as well as the hip kinematics and kinetics, did not significantly differ between the TEA group and MEA group (*p* > 0.05). At the toe-off, the TEA group was more unstable than the MEA group in the A/P direction during stair descent (−0.2451 ± 0.0264 m vs. −0.2107 ± 0.0544 m, *p*=0.029) but did not differ in the M/L direction, and the hip showed a larger internal rotator moment in TEA group after treatment comparing with the MEA group (0.0403 ± 0.0423 Nm/kg vs. 0.0079 ± 0.0267 Nm/kg, *p*=0.049). At the midstance, the TEA group showed a lower abductor moment than the MEA group (−0.6554 ± 0.1338 Nm/kg vs. −0.7744 ± 0.0956 Nm/kg, *p*=0.003).

## 4. Discussion

The purpose of our study was to investigate the efficacy of TEA for dynamic balance during stair walking in patients with KOA. We found that the effect of TEA was limited for dynamic balance in KOA patients during descent stair walking, and no more efficacious than MEA during ascent stair walking.

For KOA patients, poor balance is related to pain and muscle weakness [6]. A network meta-analysis showed that acupuncture with electrical stimulation or heat pain could relieve symptoms in most KOA patients and may be better than other acupuncture methods [27]. The main effect of EA on KOA was to ease the pain and remarkably improve physical function [28]. However, there was no significant difference in pain scores between the TEA group and MEA group in our study, and it did not agree with those reviews and two studies with similar interventions [29, 30]. One possible reason for the discrepancy is the MEA setting in acupuncture trials, which is still controversial because the nonpenetrating and superficial acupuncture needle techniques are not totally inert. Thus, MEA may have placebo effects [31]. Furthermore, acupuncture has a convergence effect, which means that its effect may be weakened with the prolongation of intervention time, and the effect will be different if the acupoints are selected differently. Plaster et al. [32] studied patients with KOA and indicated that both electroacupuncture and manual acupuncture cannot increase muscle strength. It may be the reason why TEA cannot improve the dynamic balance of patients with KOA. The main strategy used to control the gait balance is the hip strategy, as the hips play a significant role in stabilizing the COM both in the A/P and M/L directions [33]. Meta-analysis revealed that KOA patients may adopt kinematic and kinetic alterations during stair climbing, such as increasing hip/trunk flexion angle [34]. Early-stage KOA patients with knee pain may decrease their hip abductor muscle strength when negotiating stairs [35]. The results showed that there were little remarkable differences in hip angle and moments between the two groups. Exercise programmes that focused on lower limb muscle strength training were beneficial for patients with KOA to improve dynamic balance using a modified Star Excursion Balance Test [36]. Therefore, the combination of electroacupuncture and hip muscle strength training may be more effective for KOA patients to improve their dynamic balance.

Most studies [7, 8, 10] assess dynamic balance by using scales or balance tests, and fewer studies focused on dynamic balance when individuals do some daily activities, such as stair climbing. The demands for dynamic balance control during negotiation of stairs are higher than level walking. A previous study used the XCOM method to evaluate the dynamic balance for descent stair walking [16]. The dynamic stability index shows that speed is an important factor for dynamic stability assessment [37]. Slower walking velocities increase the stability [38]. Gait speed shows a strong correlative relationship with COM velocity [39]. The results of this study showed that the speed of the TEA group during ascent and descent increased after treatment. Although the difference between TEA and MEA was not statistically significant, the possible influence of speed on dynamic stability should still be considered. Due to the speed increase after treatment, the XCOM should be greater and the MOS should be less. This is in agreement with our ascent stair walking results. Even though the speed increased after treatment, there was no significant difference in the MOS values. But for descent stair walking, the MOS in the A/P direction at the toe-off event after treatment was more unstable than before its treatment. This may be related to the greater balance control needs in descent as compared with ascent [40].

This study had several limitations. One of the limitations was that all participants accepted the fixed TEA or MEA treatment. Subject-specified acupuncture treatment might be more effective for patients with KOA. Treatment according to syndrome differentiation plays an important part in traditional Chinese medicine. Acupuncture acupoints need to be selected according to the syndrome differentiation. However, it was prohibited in our study. The second limitation was that we did not assess other balance-related factors, such as muscle strength.

The third limitation was related to the use of MEA. Most of the Chinese people are familiar with acupuncture and understand the feeling of acupuncture, so it is difficult to do MEA. The position of acupoints and “de qi” sensations are the key points for acupuncture's effect. We need to deviate from the position of acupoints as much as possible and reduce the subjects' sensations to the greatest extent. Nevertheless, the multiple factors in the MEA group (depth and position of acupuncture and the electrical stimulation intensity) may have some treatment effect. In future research, these factors need to be better controlled for the MEA group.

## 5. Conclusion

Based on the assessment results from the chosen patients with KOA, the TEA did not demonstrate a significant effect in improving the dynamic balance during stair negotiation in comparison with the MEA. This finding does not support electroacupuncture as a conservative treatment to improve the dynamic balance in such patients.

## Figures and Tables

**Table 1:** The pain score and the temporal-distance, kinematic and kinetic variables of the hip during ascent.

Event	Variables	TEA group (Mean ± SD)		MEA group (Mean ± SD)		Comparisons between groups	
		Before	After	Before	After	*F* value	*P*
	Pain	5.17 ± 3.68	2.50 ± 2.20	5.83 ± 4.36	3.05 ± 2.60	0.304	0.585
	Velocity (m/s)	0.49 ± 0.09	0.52 ± 0.06	0.52 ± 0.09	0.51 ± 0.07	1.267	0.269
	Step width (m)	0.09 ± 0.03	0.08 ± 0.03	0.08 ± 0.02	0.08 ± 0.03	0.626	0.434

*Hip angle (degree, °)*							
IC	Flex (+)/Ext (−)	54.72 ± 18.95	51.71 ± 11.49	59.82 ± 12.56	56.17 ± 13.06	0.530	0.472
	Add (+)/Abd (−)	7.39 ± 4.79	6.52 ± 4.09	6.97 ± 5.17	6.34 ± 4.19	0.000	0.989
	Int (+)/Ext (−)	−5.36 ± 8.69	−2.51 ± 7.40	−6.89 ± 8.04	−2.04 ± 6.58	0.198	0.659
MS	Flex (+)/Ext (−)	27.05 ± 10.34	27.16 ± 14.15	29.54 ± 9.70	26.92 ± 8.68	0.256	0.616
	Add (+)/Abd (−)	5.79 ± 3.90	3.82 ± 3.62	3.92 ± 4.55	3.30 ± 4.58	0.569	0.45677
	Int (+)/Ext (−)	−5.11 ± 9.48	−3.41 ± 8.46	−5.94 ± 7.19	−2.21 ± 6.31	0.631	0.433
TO	Flex (+)/Ext (−)	20.50 ± 5.70	16.92 ± 7.37	19.00 ± 7.46	20.33 ± 6.26	2.789	0.104
	Add (+)/Abd (−)	−1.89 ± 4.83	−4.84 ± 3.09	−2.51 ± 4.50	−3.65 ± 3.18	2.441	0.128
	Int (+)/Ext (−)	−6.36 ± 8.14	−5.61 ± 7.13	−7.19 ± 6.78	−4.22 ± 6.83	0.688	0.413

*Hip moment (Nm/kg)*							
IC	Flex (+)/Ext (−)	−0.0495 ± 0.1346	−0.0939 ± 0.1235	−0.0226 ± 0.1360	−0.0572 ± 0.1124	0.519	0.477
	Add (+)/Abd (−)	0.0328 ± 0.0354	0.0236 ± 0.0398	0.0421 ± 0.0320	0.0317 ± 0.0400	0.194	0.663
	Int (+)/Ext (−)	0.0314 ± 0.0363	0.0346 ± 0.0375	0.0444 ± 0.0351	0.0388 ± 0.0463	0.015	0.903
MS	Flex (+)/Ext (−)	−0.3513 ± 0.2381	−0.2101 ± 0.2567	−0.1914 ± 0.1919	−0.1924 ± 0.2025	2.076	0.159
	Add (+)/Abd (−)	−0.5913 ± 0.2111	−0.5727 ± 0.2023	−0.5267 ± 0.2006	−0.6107 ± 0.1357	0.712	0.405
	Int (+)/Ext (−)	−0.2504 ± 0.1071	−0.2265 ± 0.1137	−0.2219 ± 0.0890	−0.2482 ± 0.0701	0.643	0.429
TO	Flex (+)/Ext (−)	−0.0139 ± 0.1001	−0.0093 ± 0.0659	0.0603 ± 0.0756	0.01471 ± 0.0608	0.117	0.734
	Add (+)/Abd (−)	0.0163 ± 0.1713	0.0742 ± 0.0402	0.0617 ± 0.0562	0.0823 ± 0.0504	0.232	0.633
	Int (+)/Ext (−)	−0.0060 ± 0.0763	0.0115 ± 0.0256	0.0237 ± 0.0221	0.0261 ± 0.0199	2.084	0.159

*Margin of stability (m)*							
IC	A/P direction	0.0926 ± 0.1251	0.1041 ± 0.1085	0.0851 ± 0.1234	0.1123 ± 0.1073	0.089	0.768
	M/L direction	−0.0282 ± 0.1115	−0.0546 ± 0.1020	−0.0301 ± 0.1085	−0.0625 ± 0.0914	0.057	0.813
TO	A/P direction	−0.1665 ± 0.0387	−0.1769 ± 0.0344	−0.1677 ± 0.0288	−0.1797 ± 0.0360	0.050	0.825
	M/L direction	−0.1344 ± 0.0281	−0.1390 ± 0.0290	−0.1334 ± 0.0291	−0.1380 ± 0.0317	0.004	0.947
MS	A/P direction	−0.0080 ± 0.0190	−0.0049 ± 0.0077	−0.0044 ± 0.0219	−0.0021 ± 0.0119	0.610	0.440
	M/L direction	−0.0641 ± 0.0538	−0.0462 ± 0.0606	−0.0658 ± 0.0620	−0.0522 ± 0.0613	0.077	0.783
MIN	A/P direction	−0.1642 ± 0.0396	−0.1741 ± 0.0363	−0.1637 ± 0.0343	−0.1767 ± 0.0394	0.093	0.762
	M/L direction	−0.1368 ± 0.0272	−0.1412 ± 0.0268	−0.1354 ± 0.0281	−0.1396 ± 0.0316	0.012	0.914

**Table 2:** The temporal-distance, kinematic, and kinetic variables of the hip during descent.

Event	Variables	TEA group (Mean ± SD)		MEA group (Mean ± SD)		Comparisons between groups	
		Before	After	Before	After	*F* value	*P*
	Velocity (m/s)	0.49 ± 0.09	0.53 ± 0.05	0.53 ± 0.10	0.53 ± 0.08	1.240	0.273
	Step width (m)	0.12 ± 0.03	0.11 ± 0.03	0.13 ± 0.04	0.12 ± 0.04	0.366	0.549

*Hip angle (degree, °)*							
IC	Flex (+)/Ext (−)	25.63 ± 11.54	20.68 ± 8.64	27.09 ± 7.10	24.72 ± 5.72	2.627	0.115
	Add (+)/Abd (−)	−3.23 ± 6.26	−4.82 ± 4.86	−4.50 ± 5.21	−3.77 ± 5.05	2.327	0.258
	Int (+)/Ext (−)	−8.95 ± 9.32	−9.16 ± 7.14	−10.53 ± 6.56	−6.72 ± 6.68	2.491	0.124
MS	Flex (+)/Ext (−)	20.76 ± 9.48	17.63 ± 11.16	26.19 ± 8.86	22.66 ± 7.62	0.319	0.576
	Add (+)/Abd (−)	5.02 ± 3.73	3.25 ± 2.69	4.30 ± 4.00	4.89 ± 3.86	3.123	0.086
	Int (+)/Ext (−)	−4.88 ± 9.45	−4.144 ± 9.01	−5.17 ± 7.18	−1.24 ± 7.49	1.661	0.206
TO	Flex (+)/Ext (−)	32.96 ± 11.56	31.40 ± 12.26	38.12 ± 8.42	36.91 ± 8.00	0.750	0.393
	Add (+)/Abd (−)	1.74 ± 5.16	0.36 ± 4.17	2.21 ± 4.46	1.02 ± 5.02	0.095	0.760
	Int (+)/Ext (−)	−3.28 ± 7.22	−2.15 ± 7.81	−3.21 ± 7.88	0.62 ± 6.78	1.658	0.207

*Hip moment (Nm/kg)*							
IC	Flex (+)/Ext (−)	0.0546 ± 0.0855	0.0722 ± 0.0945	0.0716 ± 0.0910	0.0909 ± 0.0750	0.047	0.829
	Add (+)/Abd (−)	0.0043 ± 0.0420	0.0142 ± 0.0375	0.0069 ± 0.0509	0.0112 ± 0.0487	0.062	0.805
	Int (+)/Ext (−)	0.0085 ± 0.0182	0.0142 ± 0.0270	0.0060 ± 0.0301	0.0090 ± 0.0224	0.320	0.575
MS	Flex (+)/Ext (−)	−0.2691 ± 0.2224	−0.1629 ± 0.2699	−0.1787 ± 0.2014	−0.1215 ± 0.2197	0.238	0.629
	Add (+)/Abd (−)	−0.7562 ± 0.1457	−0.6554 ± 0.1338	−0.7744 ± 0.2201	−0.7744 ± 0.0956	**10.572**	**0.003**
	Int (+)/Ext (−)	−0.1777 ± 0.1128	−0.1296 ± 0.1313	−0.1915 ± 0.0807	−0.1675 ± 0.0807	0.994	0.326
TO	Flex (+)/Ext (−)	0.1324 ± 0.1367	0.1473 ± 0.0962	0.2055 ± 0.0726	0.2041 ± 0.0796	2.309	0.138
	Add (+)/Abd (−)	0.0007 ± 0.0962	0.0184 ± 0.0882	0.0164 ± 0.0869	0.0162 ± 0.0614	0.111	0.742
	Int (+)/Ext (−)	0.0246 ± 0.0433	0.0403 ± 0.0423	0.0020 ± 0.0358	0.0079 ± 0.0267	**4.192**	**0.049**

*Margin of stability (m)*							
IC	A/P direction	−0.0769 ± 0.1579	−0.0728 ± 0.1604	−0.0654 ± 0.1498	−0.1088 ± 0.1419	1.009	0.323
	M/L direction	−0.0188 ± 0.1889	−0.0483 ± 0.2012	−0.0728 ± 0.2172	−0.0255 ± 0.2118	0.687	0.413
TO	A/P direction	−0.2328 ± 0.0228	−0.2451 ± 0.0264	−0.2196 ± 0.0681	−0.2107 ± 0.0544	**5.199**	**0.029**
	M/L direction	−0.2375 ± 0.0299	−0.2470 ± 0.0333	−0.2212 ± 0.0873	−0.2269 ± 0.0756	1.221	0.277
MS	A/P direction	−0.0186 ± 0.0281	−0.0270 ± 0.0365	−0.0255 ± 0.0351	−0.0243 ± 0.0365	0.292	0.593
	M/L direction	−0.0687 ± 0.0895	−0.0881 ± 0.1024	−0.0858 ± 0.1010	−0.0656 ± 0.0956	1.259	0.270
MIN	A/P direction	−0.2493 ± 0.0271	−0.2597 ± 0.0322	−0.2537 ± 0.0350	−0.2519 ± 0.0259	1.456	0.236
	M/L direction	−0.2639 ± 0.0253	−0.2765 ± 0.0237	−0.2347 ± 0.1242	−0.2726 ± 0.0262	0.317	0.577

## Data Availability

The data that support the findings of this study are available on request from the first author, Meijin Hou.
